# miR-622 Counteracts the NUAK1-Induced Gastric Cancer Cell Proliferation and the Antioxidative Stress

**DOI:** 10.1155/2022/9616764

**Published:** 2022-07-14

**Authors:** Jian Yang, Jian Lu, Ni Yin, Jingyue Sun, Jianhong Pu, Jin Zang

**Affiliations:** ^1^Department of General Surgery, The First Affiliated Hospital of Soochow University, Suzhou 215000, China; ^2^Department of Orthopedics, The First Affiliated Hospital of Soochow University, Suzhou 215000, China; ^3^Department of Oncology, The First Affiliated Hospital of Soochow University, Suzhou 215000, China; ^4^Health Management Center, The First Affiliated Hospital of Soochow University, Suzhou 215000, China; ^5^Department of Urology, The First Affiliated Hospital of Soochow University, Suzhou 215000, China

## Abstract

**Background:**

Gastric cancer (GC), a highly prevalent gastric cancer, has high-risk mortality. Thus, investigating strategies to counteract its growth is important to provide theoretical guidance for its prevention and treatment. It has been pointed out that abnormal expression of microRNAs (miRNAs) serves as noninvasive biomarkers for GC. This present study probed into the role of miR-622 and the NUAK family SNF1-like kinase 1 (NUAK1).

**Methods:**

Five mRNA datasets (GSE64916, GSE118916, GSE122401, GSE158662, and GSE159721) and one miRNA dataset (GSE128720) from the Gene Expression of Omnibus (GEO) database were used to analyze the differentially expressed miRNAs and mRNA in GC and noncancer samples. Further, western blot, real-time quantitative PCR (qRT-PCR), reactive oxygen species (ROS) assay kit experiments, and wound healing assay, together with *in vivo* experiments, were performed.

**Results:**

miR-622 was downregulated, and NUAK1 was upregulated in GC, and NUAK1 was a potential target of miR-622. Knocking down NUAK1 decreased GC cell proliferation and migration but increased oxidative stress *in vitro* and inhibited the development of tumor *in vivo*, while miR-622 acted to suppress the action of NUAK1 through the miR-622/NUAK1/p-protein kinase B (Akt) axis, thereby inhibiting the occurrence of GC.

**Conclusion:**

miR-622 and NUAK1 demonstrated potential for being targets and biomarkers for GC treatment.

## 1. Introduction

Gastric cancer (GC) is the fifth most commonly seen cancer as well as the third most general cause of cancer death around the globe [1]. It is also one of the most commonly existing malignant tumors in the digestive system, with a mortality rate of about 80% [2]. Gastric adenocarcinoma (GA), being the main histological type of cancer, occupies 95% of all GCs [3]. Although much medical progress has been made and the incidence is declining, less than 30% of cancer patients can survive ≧5 years [4]. Due to its few alarming symptoms during its early stage, it is often diagnosed when it has already reached a late stage, thus leading to few treatment choices and poor prognoses [5, 6]. Therefore, finding new and effective diagnostic methods is important to better understand this disease. Recently, there are studies showing that oxidative stress could serve as a potential strategy of cancer treatment, a process that exerts an effect in the pathogenesis for multiple stomach diseases, while GA, as a malignant tumor disease, progresses by a variety of regulators, including microRNA, which acts as an important regulator in cancer and other pathology [7, 8].

MicroRNA (miRNA) is 20-24 or so nucleotides in length of small RNA and has varieties of significant regulatory functions in cells [9]. It participates in the posttranscription regulation of gene expression in multicellular organisms via influencing the translation and stability of mRNA [10]. There can be multiple target genes in each miRNA, and a few miRNAs are able to regulate the same gene. The above complex regulatory network is capable of regulating the multiple gene expression either by a sole miRNA or via a combination of miRNA. However, the role of miRNA in GA is unclear.

More and more evidence indicates a tumor suppressor role of miR-622 in several types of human cancer, such as pancreatic, glioma, hepatocellular, and others, affecting cell proliferation, migration, and metastasis [11–13]. Besides, dual-specificity tyrosine-(Y)-phosphorylation regulates kinase 2 (DYRK2) and miR-622 inhibit the invasion and migration of colorectal cancer cells by targeting the Kirsten rat sarcoma viral oncogene homolog (KRAS) [14]. miR-622 can inhibit cancer metastasis in lung cancer via suppressing Hypoxia-inducible Factor 1*α* (HIF-1*α*) [15]. miR-622 targets Yes-associated protein (YAP) in glioma and targets ring finger protein 8 (RNF8) in breast cancer [16, 17].

In spite of the increasing evidence on the role of miR-622 in human tumorigenesis, its function in GA is not fully understood. Therefore, this study intended to screen differentially expressed genes through bioinformatics methods, verify the signal pathway through experiments, and identify molecular targets with biological significance to provide theoretical support for the study of GC pathogenesis.

## 2. Materials and Methods

### 2.1. Gene Expression Omnibus Series (GSE) Dataset Screening

First, the keyword “gastric adenocarcinoma” was retrieved from the Gene Expression Omnibus (GEO) database. Between healthy and diseased tissues, the screening conditions were set as the control. The data sets were selected, cleaned, and annotated. The screening conditions of differentially expressed genes were *P* < 0.05 and |logFC| > sum(abs(GSE logFC))/length(GSE logFC). Finally, the intersection analysis of multiple data sets was conducted.

### 2.2. Protein-Protein Interaction (PPI) Network Analysis

The database search tool STRING (https://string-db.org/) for biological prediction network using searchable gene interactions analyzed and evaluated the interactions between differentially expressed genes (DEGs). The combination score > 0.4 was designed to be a criterion of cut-off. The PPI data obtained by STRING platform analysis was imported into the Cytoscape software, and CytoHubba was used to screen the core genes.

### 2.3. Cell Culture and Grouping

The culture of AGS cells bought from Procell Life Science & Technology Co., Ltd. (Wuhan, China) was performed in 5% CO_2_ at 37°C with 10% FBS/RPMI-1640 (C11875500BT, 26010074, Grand Island, NY, USA) medium. They were divided into the NC-shRNA group, the NUAK1-shRNA group, and the NUAK1-shRNA+miR-622 inhibitor. miR-622 inhibitor, which was used to suppress miR-622 expression, was purchased from GenePharma (Shanghai, China).

### 2.4. Design and Preparation of NUAK1-shRNA

pLKO.1-EGFP-puro-NUAK1-shRNA plasmid and control plasmid NC-shRNA were devised and synthesized by Guangzhou All-perfect Biological Technology Co., Ltd (Guangzhou, China). When the AGS cells reached 60-80% confluence, the Lipofectamine™ RNAiMAX (13778150, Carlsbad, CA, USA) was employed to make a transfection of the cells following the manufacturer's instructions. The highest interference shRNA was selected for subsequent lentivirus packaging and a 1 × 10^8^ TU/mL titer. Then, NUAK1-shRNA-AGS cell lines were screened by a fluorescence microscope.

### 2.5. Reactive Oxygen Species (ROS) Analysis

The ROS level was measured according to the method described previously [18], with some modifications. Briefly, the reactive oxygen species assay kit (S0033, Beyotime, Shanghai, China) was applied to detect the change in ROS content following the instructions of the manufacturer. DCHF-DA was utilized to stain the cells (1 : 3,000) at 37°C for 30 min. The oxidized DCF has a maximum emission at 525 nm and was analyzed using flow cytometry (BD Biosciences, CA, USA).

### 2.6. Wound Healing Assays

Inoculation of cells (1 × 10^5^ cells/well) were performed in 24-well plates. After 24 h starvation culture, a medium containing 10% FBS took the place of the origin medium. Wounds were formed with a plastic tip through the monolayer of cells. PBS was utilized to wash cells. Wound closure was observed under a microscope at 0 h and 24 h, separately. The area covered by cell migration (%) was quantified by ImageJ. All experiments were performed in 3 replicates.

### 2.7. Real-Time Quantitative PCR (qRT-PCR)

Extract of total RNA was obtained from cancer cells utilizing the TRIzol reagent (Invitrogen, CA, USA), and its concentration was determined. Reverse transcription of the RNA was conducted by the PrimeScript RT Reagent Kit (Takara, Tokyo, Japan) based on the manufacturer's instructions. The Bestar™ qPCR-RT-Kit (DBI-2220, DBI Bioscience, Ludwigshafen, Germany) was applied to the cDNA synthesis by referring to the manufacturer's instructions. QRT-PCR was performed using Bestar® SYBRGREEN qPCR Mastermix (DBI-2043, DBI Bioscience). The primers used are shown in Table 1. Each reaction was repeated three times.

### 2.8. Western Blotting

Protein studies were performed in accordance with standard procedures. Anti-NUAK1 and p-Akt antibodies were bought from Cell Signaling Technology (Beverly, USA). From Bio-Rad, secondary anti-mouse and anti-rabbit antibodies coupled to horseradish peroxidase were collected. Through an enhanced chemiluminescence detection kit (Thermo Fisher Scientific, Waltham, MA, USA), enhanced chemiluminescence visualization was obtained.

### 2.9. Animal Model Establishment and Intervention Methods

Six specific pathogen-free (SPF) male BALB/c-nu mice (aged 4 weeks and 14–16 g) were bought from SiPeiFu Biotechnology Co., Ltd (Beijing, China). All the animals were randomly distributed into two groups (*n* = 3 mice/group) after one-week adaptive feeding. Different types of AGS cell resuspension solution were injected into the left axillary area of nude mice at a concentration of 1.5 × 10^7^ cells 100 *μ*L. The NC-shRNA group was injected with NC-shRNA-AGS cell resuspension solution, and the NUAK1-shRNA group was injected with NUAK1-shRNA-AGS cell resuspension solution. Subcutaneous nodules were observed weekly after implantation, and the diameter of each nodule was measured and recorded. When the diameter of the subcutaneous nodule reached >0.3 mm and was hard and fixed, this meant that the tumor-bearing model was successfully established, and the drug intervention could be performed. Twenty-eight days after injection, the mice were euthanized, and the examination of subcutaneous growth of each tumor was carried out. The study was performed strictly in line with the National Institutes of Health's Animal Care and Use Guidelines. The animal experiment was conducted following the guidelines of the committee of animal research institutions, which is in conformity to the national guidelines for the care and use of experimental animals.

During the experiment, the width and length of the tumor were measured with a vernier caliper every week, using the following formula: *V* (tumor volume, cm^3^) = 0.52 × *L* × *W*2, where *L* refers to the maximum length of tumor block and *W* the maximum width perpendicular to the maximum diameter.

### 2.10. Statistical Analysis

The R 4.0.6 (http://www.rstudio.com/products/rstudio) software and GraphPad Prism 9.0 (La Jolla, CA) software were employed for statistical analyses. All data was represented as mean ± standard deviation (SD). The unpaired Student *t*-test was applied to two-group comparison. And when more than two groups were evaluated, one-way analysis of variance (ANOVA) as well as Tukey's multiple comparison test was adopted. *P* < 0.05 was considered as significant difference.

## 3. Results

### 3.1. mRNA Datasets and Screening of Differentially Expressed Genes

**Figure 1:**
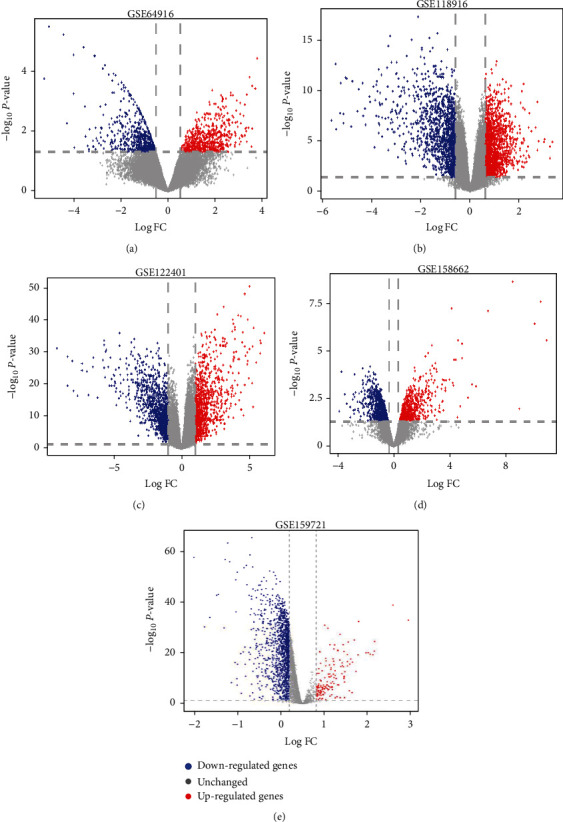
Volcano map of differentially expressed genes in five mRNA gastric cancer databases: (a) GSE64916; (b) GSE118916; (c) GSE122401; (d) GSE158662; (e) GSE159721. The red, blue, and gray dots represent upregulated genes in gastric cancer, downregulated genes, and unchanged genes, respectively.

We searched from the GEO database using “gastric carcinoma” as the keyword. The dataset contained gastric cancer and noncancer samples. A total of 5 mRNA datasets (GSE64916, GSE118916, GSE122401, GSE158662, and GSE159721) and one miRNA dataset (GSE128720) were identified (Figure 1 and Table 2).

**Figure 2:**
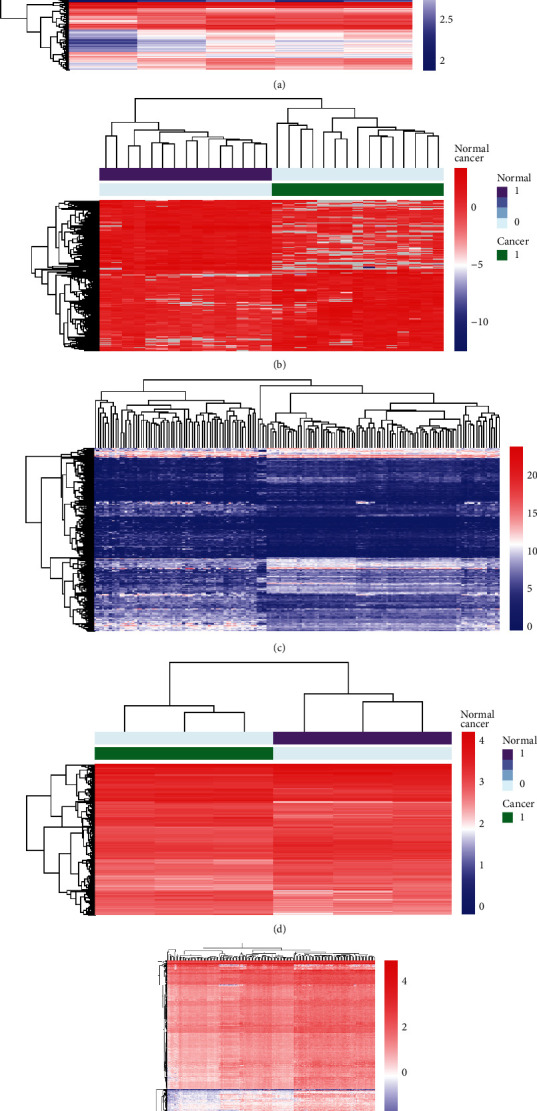
Cluster heatmaps of differentially expressed genes in five mRNA datasets: (a) GSE64916; (b) GSE118916; (c) GSE122401; (d) GSE158662; (e) GSE159721. Red indicates relatively upregulated genes in gastric cancer; blue represents downregulated genes.

Quality-controlled RNA-seq data was subjected to differential analysis. The GSE64916 dataset screened 1343 differential genes, and among them, 720 genes were upregulated while 623 were downregulated. A total of 2862 differential genes were screened in the GSE118916 dataset, with 1544 genes upregulated and 1318 genes downregulated. For the GSE122401 dataset, of the 2227 differential genes identified, 1175 were upregulated and 1052 were downregulated. For the GSE158662 dataset, of the 1693 differential genes screened, 718 were upregulated and 975 were downregulated. Lastly, for the GSE159721 dataset, of the 1480 differential genes screened, 153 were upregulated and 1327 were downregulated (Figures 1(a)–1 (e) and 2(a)–2 (e)).

### 3.2. Pathway Analysis of the mRNA Datasets

**Figure 3:**
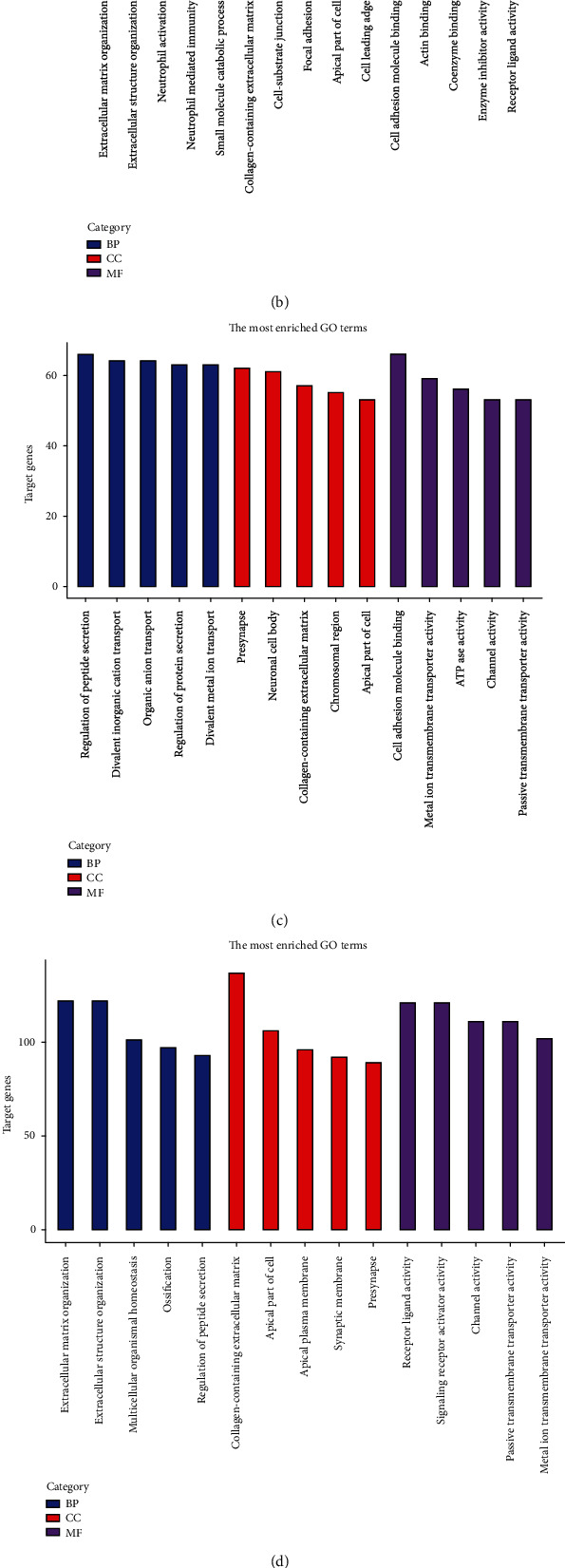
GO enrichment analysis of differentially expressed genes: (a) GSE64916; (b) GSE118916; (c) GSE122401; (d) GSE158662; (e) GSE159721. BP: biological process; CC: cellular component; MF: molecular function.

**Figure 4:**
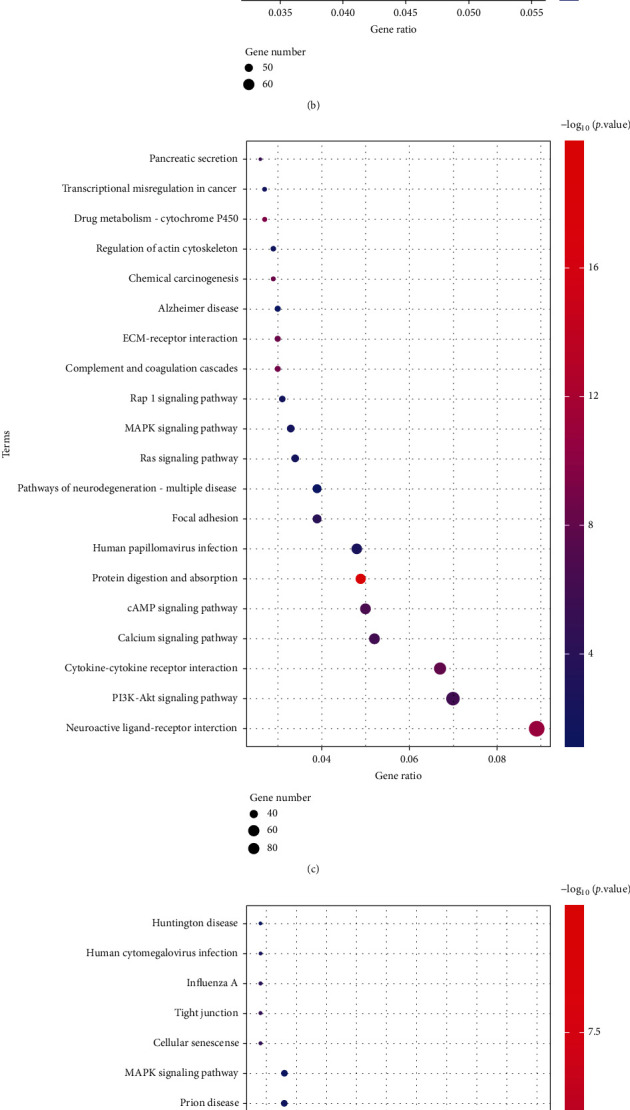
KEGG enrichment analysis of differentially expressed genes: (a) GSE64916; (b) GSE118916; (c) GSE122401; (d) GSE158662; (e) GSE159721.

Gene Ontology (GO) and Kyoto Encyclopedia of Genes and Genomes (KEGG) enrichment analyses were carried out on the differential genes screened in the 5 datasets. Among them, GO was divided into three portions, namely, cellular component (CC), molecular function (MF), and biological process (BP). As results have shown, the GSE64916 dataset was principally abundant in the collagen-containing extracellular matrix and PI3K-Akt signaling pathway. The GSE159721 dataset was in the main rich in cell adhesion molecule binding and human papillomavirus infection. The GSE118916 dataset was chiefly enriched in the collagen-containing extracellular matrix and pathways of neurodegeneration-multiple diseases. The GSE158662 dataset was mainly enriched in organelle fission and the PI3K-Akt signaling pathway. The GSE122401 dataset appeared plentiful primarily in the collagen-containing extracellular matrix and neuroactive ligand-receptor interaction (Figures 3(a)–3(e) and 4(a)–4(e)).

### 3.3. Core Gene Screening

**Figure 5:**
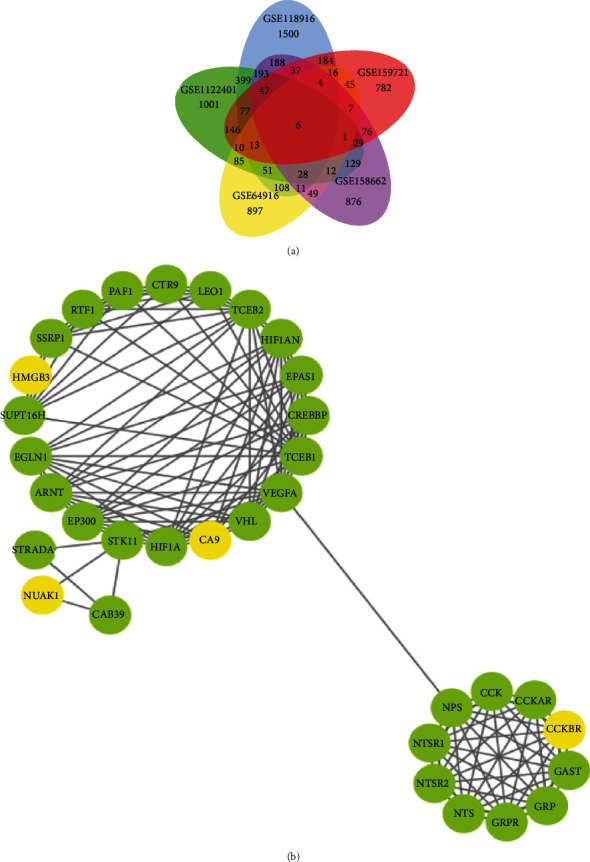
Expression analysis of core genes and PPI network analysis: (a) Venn diagram of differentially expressed genes in the five mRNA datasets; (b) construction of protein interaction network. Green represents the genes clinically closely related to the occurrence of gastric adenocarcinoma derived from the STRING database, and yellow represents the four key genes identified.

The five mRNA datasets were intersected with significantly different genes. Six core genes were obtained, namely, Carbonic Anhydrase IX (CA9), Cholecystokinin B Receptor (CCKBR), Beta-1,3-Glucuronyltransferase 1 (B3GAT1), Mesoderm Specific Transcript (MEST), NUAK1, and high mobility group box protein 3 (HMGB3) (Figure 5(a) and Table 3).

### 3.4. PPI Network Analysis for Identifying Key Genes

The 30 genes related to NACLC obtained from the STRING database were combined with 6 core genes, combined with Cytoscape for protein interaction network analysis, and 4 key genes were screened, namely, HMGB3, CA9, NUAK1, and CCKBR (Figure 5(b)).

### 3.5. Target Gene Prediction

**Figure 6:**
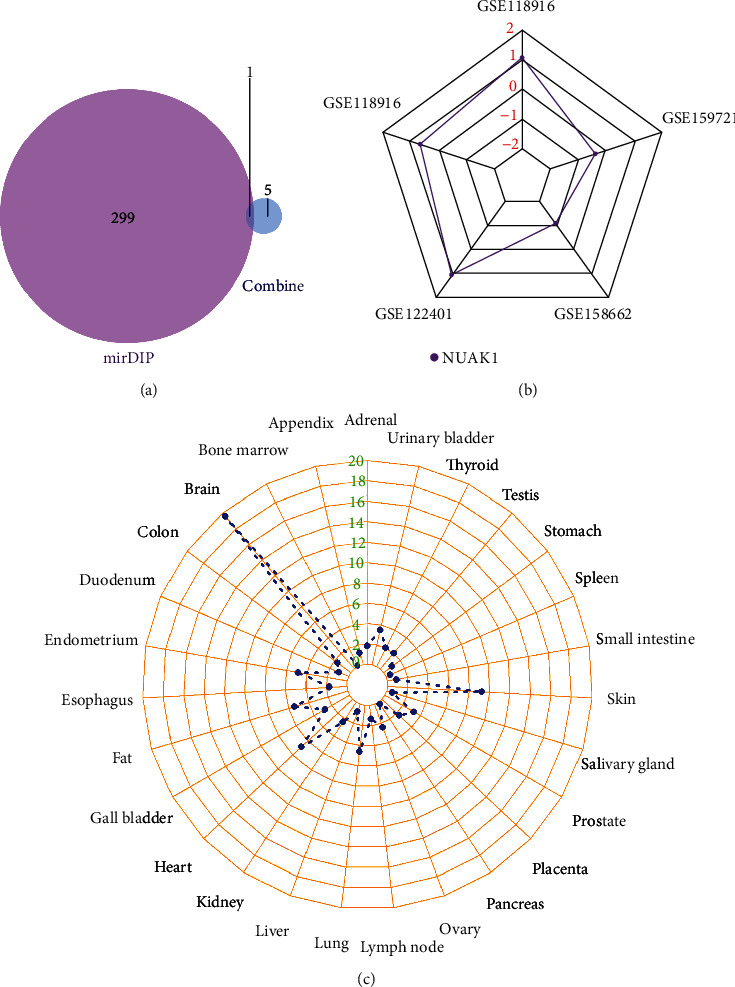
Target gene prediction results. (a) miRDIP target gene prediction database predicts the target genes of hsa-miR-622 and hsa-miR-6872-5p. One target gene is NUAK1; (b) NUAK1 logFC value in the five mRNA datasets. (c) The expression level of NUAK1 in different organs and tissues.

The threshold logFC value of miRNA dataset GSE128720 was 2.9, and 2 differential genes were screened, of which none were upregulated genes, and two were downregulated genes (hsa-miR-622 and hsa-miR-6872-5p). The miRDIP target gene prediction database was employed to predict the target genes of hsa-miR-622 and hsa-miR-6872-5p, 300 mRNAs were identified, and at the same time, the six core genes obtained in this study were intersected, which identified one target gene: NUAK1 (Figure 6(a)). NUAK1 was the miR-622 target gene and showed an upregulation trend in the dataset (Figure 6(b)). Additionally, the expression was shown to be the highest in the brain by the results (Figure 6(c)).

### 3.6. Significance of NUAK1 and miR-622 Regulatory on Cell Migration and Oxidative Stress

**Figure 7:**
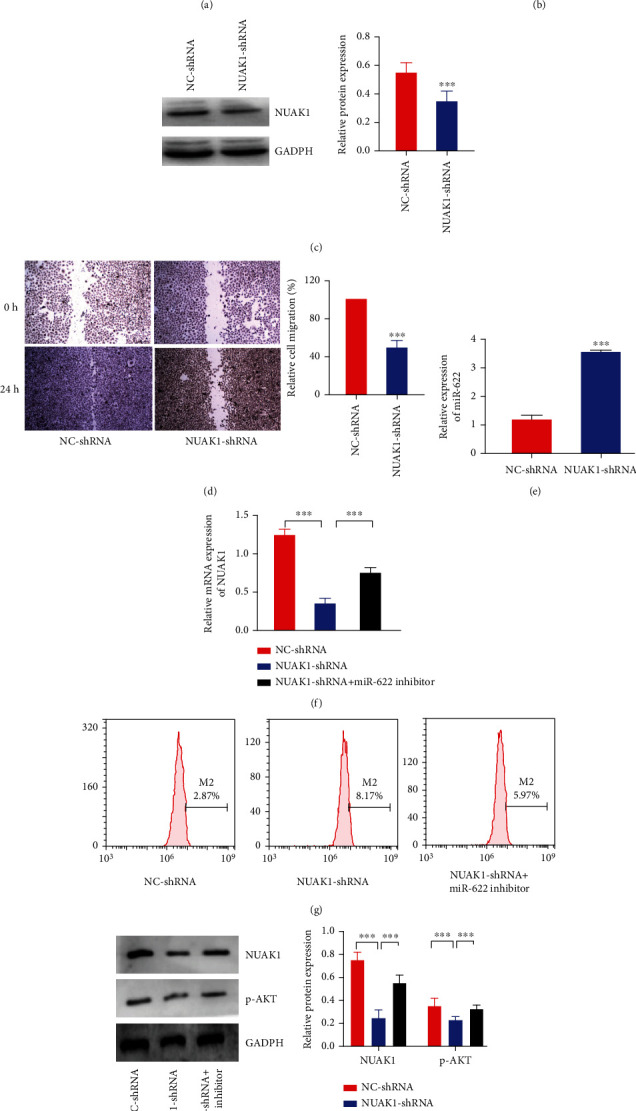
NUAK1/miR-622 axis regulates cell migration ability and oxidative stress. (a) NUAK1-shRNA screening. After transfection of three shRNAs, a fluorescence microscope was adopted to detect the content of NUAK1 in AGS cells. (b, c) Expression of NUAK1 mRNA (b) and protein (c) after transfection with NUAK1-shRNA was detected by qRT-PCR and western blot. (d) The migration ability was assessed by wound healing assay. (e) Expression of miR-622 after transfection with NUAK1-shRNA was tested by qRT-PCR. (f) The expression of NUAK1 was measured by qRT-PCR after transfection with NUAK1-shRNA and miR-622 inhibitor. (g) Flow cytometry for ROS activity. (h) The expression of NUAK1 and p-AKT was examined by western blot. ^∗∗∗^*P* < 0.001.

After transfection of shRNA, the content of NUAK1 in AGS cells was detected, which showed that NUAK1-shRNA was successfully transfected (Figure 7(a)). And the protein expression of NUAK1 and mRNA was decreased in AGS cells after transfection of NUAK1-shRNA plasmid (Figures 7(b) and 7(c)). These results confirmed that NUAK1 expression was successfully knocked down. The migration ability of AGS was found to decrease significantly after silencing NUAK1 when contrasted with the NC-shRNA group (*P* < 0.001, Figure 7(d)).

**Figure 8:**
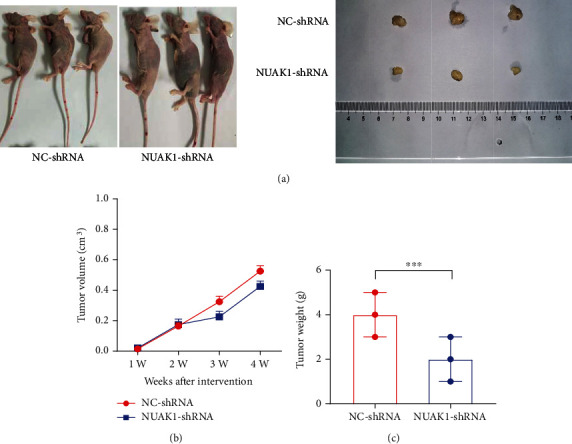
Effect of NUAK1 knockdown on tumor growth *in vivo* experiments. (a) Images of representative tumors excised from mice. (b) Tumor volume reflects tumor growth curve. (c) The changes of tumor weight of mice. ^∗∗∗^*P* < 0.001.

Apart from the above, the expression of miR-622 was evaluated utilizing qRT-PCR. The data (Figure 7(e)) exhibited a high expression of miR-622 in the NUAK1-shRNA group which was upregulated in comparison with the NC-shRNA group. Moreover, the results in Figure 7(f) revealed that interference with miR-622 expression could derepress the inhibitory effect of NUAK1-shRNA on NUAK1 expression (*P* < 0.001). The change of ROS content in the AGS cells per group is shown in Figure 8(g). The data displayed that the ROS content increased after NUAK1 knockdown when making a comparison with the NC-shRNA group (*P* < 0.001), while the addition of the miR-622 inhibitor to the NUAK1-shRNA group decreased the ROS content (*P* < 0.0001). It could be observed that the protein expression of p-Akt and NUAK1 was decreased by knocking down NUAK1 (*P* < 0.001) when it was contrasted with the NC-shRNA group, and meanwhile, treatment with the miR-622 inhibitor reversed the effects of the NUAK1silencing (Figure 7(h)).

### 3.7. Significance of NUAK1-Regulated In Vivo Settings

Finally, we confirmed the effect of NUAK1 expression on the growth of tumor by *in vivo* experiments. The mice's tumor growth changes in each group are shown in Figure 8(s). In terms of phenotype, the growth rate of tumor volume (Figure 8(b)) and weight (Figure 8(c)) in the NUAK1-shRNA group decreased in comparison with the NC-shRNA group (*P* < 0.001). Thus, our finding showed that NUAK1 knockdown inhibited the tumor growth and proliferation.

## 4. Discussion

Tumor cells are often exposed to oxidative stress in a variety of environments in the body. The tumors quickly grow from blood supply, resulting in hypoxia, which is frequently supported through stimulating angiogenesis. Nevertheless, chaotic blood flow within new blood vessels is responsible for intermittent hypoxia, which is then filled. This refibrillation causes the production of reactive oxygen, and it may attribute to oxidative stress in tumors [19]. At this time, the increase of tumor metabolic pressure also enhances the production of reactive oxygen, affecting downstream signals as well as inducing cell death [20]. Oxidative stress is involved in cell carcinoma by inducing DNA mutations, which promote cancer progression. At the same time, the progression of GA is mediated by multiple regulators, covering miRNA [21, 22]. In recent years, the application of bioinformatics in medical molecular biology led to considerable repercussions, greatly improving the efficiency of clinical treatment and basic research content [23, 24]. Using bioinformatics, this study identified a target gene, NUAK1, and a miRNA, miR-622, from tens of thousands of alternative genes as key indicators of the GA. Our initial analysis showed that NUAK1, the target gene for miR-622, concurred with the results of Orlandella et al. [25].

Among the human adenosine monophosphate-activated protein kinase family, NUAK1 is one member of them. It has been found to be expressed in various human malignancies and is believed to be linked to tumor metastasis as well as invasion. It has also been shown to participate in multiple processes such as cell multiply, cell adhesion, aging regulation, tumor progression, and cell proliferation [26]. Previous literature has linked the overexpression of NUAK1 to the overall survival and disease-free survival of GC patients, indicating NUAK1 as a valuable molecular biomarker of GC [27]. Furthermore, NUAK1 not only is a key component of antioxidant defense systems but also is vital to tumor survival [28]. In one experiment, it was found that NUAK1 was associated with decreased oxidative stress in tumors; thus, we hypothesized that increased expression of NUAK1 could potentially alleviate oxidative stress processes, reduce the production of reactive oxygen, and hinder tumor cell apoptosis [29]. miR-622 is a short noncoding RNA that participates in the posttranscription regulation of gene expression in multicellular organisms via influencing the translation and stability of mRNA [30–32]. In this present study, our data demonstrated that the miR-622's expression was upregulated, and p-Akt and NUAK1's expressions were downregulated after the NUAK1 gene was knocked down compared with the NC-shRNA group.

Previous studies showed that an overexpression of miR-622 was related to decreasing risks of various tumors. In this study, according to bioinformatics analysis, miR-622 belongs to the downward gene in GA cells, so it can be inferred that miR-622 exerts an anticancer effect in GA, but related studies were missing. We initially speculated on the results predicted through our online website that miR-622 could inhibit the expression of NUAK1. The inverse relationship between NUAK1 and miR-622 observed in this study concurs with that reported by Orlandella et al. [25]. These suggest that one of the roles of miR-622 could be to maintain the expression level of NUAK1 within a physiological range in normal tissues.

The 3′UTR of NUAK1 has been shown to be targeted by multiple deregulated miRNAs in several cancers. For instance, Yu et al. and Shi et al. showed that miR-204 acted as a tumor suppressor through suppressing NUAK1 expression in liver cancer and non-small-cell lung carcinoma, respectively [33, 34]. Obayashi et al. reported that miR-203 demonstrated a tumor-inhibitory function in invasion and EMT induction in head and neck squamous cell carcinoma by targeting NUAK1 [35]. Further, reactive oxygen species (ROS) have been shown to affect cancer evolution contradictorily, such as either initiating/motivating cancerogenesis and backing cancer cell transformation/proliferation or leading to cell death [36]. Tumor cells can alter their metabolisms in order to adapt to high ROS level, such as NADPH generation, sulfur-based metabolism, and antioxidant transcription factor activities [37]. Based on the findings of this present study, we reasoned that oxidative stress produces reactive oxygen, which causes apoptosis of tumor cells and inhibits GA. Silencing NUAK1 increases the release of ROS and prevents tumor cell replication. miR-622 inhibited the GA cancer cell growth occurrence of GC by inhibiting the expression of NUAK1 and counteracting its cancer-promoting effects. These findings suggest that NUAK1 could be an fascinating marker of predicting malignant behavior in cancer, and could be targeted by several miRNAs which in turn could be used for making therapeutic drugs that could combat overexpression of NUAK1 and prevent cancer progression.

## 5. Conclusion

Using bioinformatics, we identified NUAK1 and miR-622 as significant differential expression genes for GC. Adopting *in vitro* and *in vivo* settings, we showed that knocking down NUAK1 promoted the cell migration ability of GC cell line, was correlated to a decrease in tumor growth and rise on ROS content, while miR-622 tended to counteract the activities of NUAK1. Further, preliminary mechanistic studies showed that these could possibly occur through the miR-622/NUAK1/p-Akt axis. Thus, our research found that NUAK1 and miR-622 could serve as potential biological targets for GC.

## Figures and Tables

**Table 1:** Sequence list of target gene primers.

Gene	Forward primer (5′->3′)	Reverse primer (5′->3′)
NUAK1	CTGAGGTCATGCTAGAGCGG	TGTCCAACAGCTCCGAAGAC
miR-622	GCGAGATCTGAGGAAGTAAAAGGCTTACAAG	GCGCTCGAG GCTTGACCTTGATGTTCAGCAGG
p-Akt	CAGATGATGCCAAGGAGATT	TGGTCAGGAGGAGTGATTGT
GADPH	CCGCGAGTACAACCTTCTTG	CAGTTGGTGACAATGCCGTG

**Table 2:** Dataset information.

Dataset	Number of samples		GPL information	Type
	Tumor	Normal		
GSE64916	4	1	GPL13497 Agilent-026652 Whole Human Genome Microarray 4x44K v2 (probe name version)	mRNA
GSE118916	15	15	GPL15207 [Prime View] Affymetrix Human Gene Expression Array	mRNA
GSE122401	80	80	GPL16791 Illumina HiSeq 2500 (Homo sapiens)	mRNA
GSE158662	3	3	GPL22755 Agilent-076500 Human lncRNA + mRNA array (probe name version)	mRNA
GSE159721	123	123	GPL20795 HiSeq X Ten (Homo sapiens)	mRNA
GSE128720	3	4	GPL24741 Agilent-070156 Human_miRNA_V21.0_Microarray 046064 (gene name version)	miRNA

**Table 3:** LogFC values of core genes.

Gene	DEG64916logFC	DEG118916logFC	DEG122401logFC	DEG158662logFC
CA9	1.1041131	-3.713271494	-3.006948077	4.759611281
CCKBR	-1.8707708	-3.670607315	-5.705510796	3.428231585
B3GAT1	0.84762935	-0.966430735	-3.594222579	2.299551681
MEST	1.978425	1.020688186	1.734825068	-0.524011847
NUAK1	1.05918085	0.638100559	1.077784907	-1.084291923
HMGB3	-1.0070922	0.737065076	1.034615104	-0.811162281

## Data Availability

The data used to support the findings of this study are available from the corresponding author upon request.
